# A High-Sensitivity Methane Sensor with Localized Surface Plasmon Resonance Behavior in an Improved Hexagonal Gold Nanoring Array

**DOI:** 10.3390/s19214803

**Published:** 2019-11-05

**Authors:** Hai Liu, Cong Chen, Yanzeng Zhang, Bingbing Bai, Shoufeng Tang

**Affiliations:** 1 School of Information and Control Engineering, China University of Mining and Technology, Xuzhou 221116, China; lhai_hust@hotmail.com (H.L.); TS18060003A31@cumt.edu.cn (C.C.); TS18060020A31@cumt.edu.cn (Y.Z.); TS18060001A31@cumt.edu.cn (B.B.); 2 Xuzhou Key Laboratory of Artificial Intelligence and Big Date, Xuzhou 221116, China

**Keywords:** localized surface plasmon resonance, methane sensor, nanorings array

## Abstract

This paper proposes a methane sensor based on localized surface plasmon resonance (LSPR) of a hexagonal periodic gold nanoring array. The effects of structural parameters on the extinction spectrum and refractive index (RI) sensitivity are analyzed to obtain optimal parameters. In particular, the RI sensitivity can reach 550.08 nm/RIU through improvement of the sensor structure, which is an increase of 17.4% over the original value. After coating a methane-sensitive membrane on the inner and outer surfaces of the gold rings, the methane concentration can be accurately measured with a gas sensitivity of −1.02 nm/%. The proposed method is also applicable to quantitative analyses of components concentration and qualitative analyses of gas composition.

## 1. Introduction

Surface plasmon polariton (SPP) [1,2,3,4,5] is a surface free-electron oscillation existing at the interface between two materials with a dielectric constant of the opposite sign. Such an evanescent wave propagates along a metal surface through free oscillating electrons that interact with photons. The localized surface plasmon resonance (LSPR) behavior [6,7,8,9] is a free-electron oscillation that is usually confined to metal nanostructures. The essence of the LSPR phenomenon is that the incident light wave transfers into the surface plasmon resonance in the form of an evanescent wave. In comparison to an SPR-based sensor, the LSPR-sensing device can be simpler. High-precision LSPR measurements can be achieved by the use of a miniature light source and a spectrometer. Recently, researchers have conducted in-depth research on different LSPR-sensing structures, including nanospheres [10], nanorings [11,12], and nanoroads [13]. Most of these structures are composed of periodic rectangular arrays. LSPR is also used in other fields [14,15,16]. In 2018, Wang et al. designed a periodic gold nanoring array for refractive index (RI) measurements with a sensitivity of 544 nm/RIU [17]. In the same year, Viswanath et al. focused on the influence of the array sequence of gold nanopores on sensing performance [18]. The RI sensitivity was found to be about 470.49 nm/RIU. Relatively speaking, the coupling strength of hexagonal periodic gold nanorings is larger than that of other sensor structures with much simpler fabrication processes. This provides us with a new means for solving some difficult problem related to the measurement of gas by optical sensing.

The reported optical gas sensors are mainly based on the optical absorption spectroscopy technology, and the greatest challenge lies in enhancing the gas sensitivity and simplifying the sensor structure. In 2018, Liu et al. designed a simpler PCF-SPR structure for simultaneous measurements of hydrogen and methane with high sensitivity [19]. However, the coating process and gas diffusion in the PCF structure is relatively difficult, which in turn greatly affects the gas detection efficiency. Therefore, the combined use of LSPR and gas-sensitive films is proposed to solve this problem. In this study, we adopted an improved hexagon gold ring array in order to enhance the refractive index sensitivity and response speed. In 2016, J. Yang et al. studied the response speed of methane-sensitive films [20]. When the methane-sensitive-film thickness is 240 nm, the response time is approximately 60 s. Meanwhile, the desorption time following spectral detection is about 180 s. In our simulations, and with the coating thickness set at *t* = 8 nm, the response speed could have been faster. This is because the resonance enhancement was influenced not only by the dipole resonance of the single nanoring, but also by the coupling between different nanorings. Finally, the refractive index sensitivity reached a maximum of 550.08 nm/RIU, which was a 17.4% increase from the original value, and the methane gas sensitivity reached −1.02 nm/%. In contrast to reported gas sensors [21,22,23], the coating process was simpler, and the sensor structure was conducive to an enhancement in the gas sensitivity for rapid concentration measurements.

## 2. Sensing Principles and Model Optimization

The incident light excites LSPR when the wave vector condition is satisfied, and the resonance position is determined by the geometry, size, and surrounding medium of the nanoparticles. Equation (1) represents the dispersion relationship of the LSPR, where l is the depth of penetration, and $\varepsilon_{1}$ and $\varepsilon_{2}$ are the dielectric constants of the metal and the medium, respectively. The resonance frequency is determined by Equation (2), where $\omega_{p}$ is the angular frequency of the surface plasmons. The extinction peak of LSPR depends on the dielectric environment around the particle, and Equation (3) describes the frequency-dependent analytical form of the dielectric constant $\varepsilon_{1}$. This form is based on the widely adopted Drude model, where γ is the damping coefficient of the material. In visible or near-infrared wavelengths, Equation (3) can be written as Equation (4) because $\gamma \langle \langle \omega_{p}$. The resonance is generated at $\varepsilon_{1}=2\varepsilon_{m}$, and Equations (5) and (6) denote the resonance frequency, $\omega_{\max}$, and the resonance wavelength, $\lambda_{\max}$, respectively. In these equations, $\varepsilon_{m}$ is the surrounding dielectric constant, and $\varepsilon_{m}=n_{m}^{2}$, $\lambda_{p}$ is the isochronous elementary frequency of the corresponding bulk metal. Therefore, the LSPR peak wavelength and the refractive index can be approximately regarded as linear in the optical band.

(1) $$\frac{\varepsilon_{1}}{\varepsilon_{2}}+\frac{l+1}{l}=0$$

(2) $$\omega_{s}=\omega_{p}\sqrt{\frac{l}{\varepsilon_{s}(l+1)+1}}$$

(3) $$\varepsilon_{1}=1-\frac{\omega_{p}^{2}}{\omega^{2}+\gamma^{2}}$$

(4) $$\varepsilon_{1}=1-\frac{\omega_{p}^{2}}{\omega }$$

(5) $$\omega_{\max}=\frac{\omega_{p}}{\sqrt{2\varepsilon_{m}+1}}$$

(6) $$\lambda_{\max}=\lambda_{p}\sqrt{2n_{m}^{2}+1}$$

**Figure 1:**
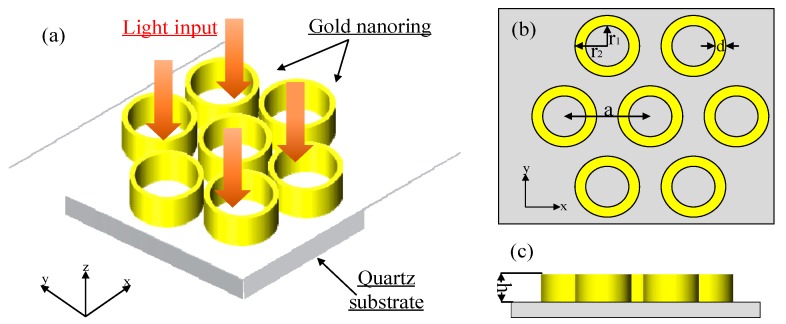
Structure of the gold nanoring array: (**a**) 3D view, (**b**) top view, and (**c**) side view.

Figure 1a shows that the LSPR sensor structure (Structure A) consists of a hexagonal periodic gold nanoring array that is located above the quartz substrate. The gold nanoring is prepared by photolithographic-patterning electrodeposition, and the quartz substrate is only used as a preparation carrier for the gold nanoring. The nanoring arrays exhibit periodic distribution in both the *x*- and the *y*- directions. Figure 1b,c indicate the main parameters of the sensor structure, including the inner radius $r_{1}$, the outer radius $r_{2}$, the lattice constant a, and the height h. In the simulation model, the background index is set at 1, while the mesh accuracy is set at 2 by default. For boundary conditions, we selected a periodic mode in *x*- and *y*-directions and a PML mode in z-direction. The source shape was a plane wave, and the plane wave type was BFAST. The incident light was perpendicular to the nano-array and is indicated by the red arrows in Figure 1a. The transmittance *T* and reflectance *R* were obtained through wavelength scanning and intensity detection. Then, the extinction coefficient E(λ)=1−T(λ)−R(λ) was calculated. The sensing characteristics were evaluated by analyzing the resonant peak-shift in the extinction spectra in relation to the background refractive index variation under different parameters.

First, the effects of thickness, height, and period on the extinction spectrum were analyzed. In the analysis of the influence of thickness, the radius of the outer ring varied from 110 nm to 150 nm with a step size of 10 nm. Since the electric field between different gold rings can be negligible when the period is a = 400 nm, the resonance was caused by the LSPR of single nanorings. As shown in Table 1, the resonance peak, full width at half maximum (FWHM), and extinction rate for different values of the parameters were obtained. The extinction rate can be obtained by the ratio of extinction to incident light. Obviously, when a = 320 nm, h = 150 nm, and d = 40 nm, the extinction rate can reach a maximum of 97.2%, while FWHM is only 64 nm. High intensity and narrow linewidth mean that such LSPR sensors have higher wavelength resolution and sensitivity. However, it was still necessary to investigate the influence of each parameter on RI sensitivity to obtain the optimal combination of parameters. Here, the refractive index sensitivity can be obtained by analyzing the displacement of the coupling peak in the unit refractive index. The background RI changed from 1.34 to 1.37 with a step size of 0.01 [24].

**Figure 2:**
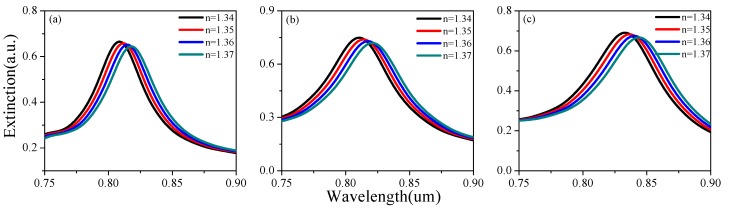
The effect of gold nanoring thickness (*t*) on the extinction spectrum under different refractive indices (RI): (**a**) d = 20 nm, (**b**) d = 30 nm, and (**c**) d = 40 nm.

**Figure 3:**
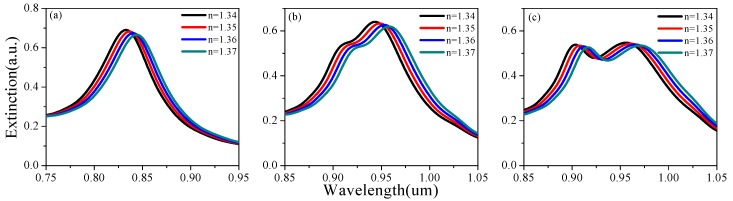
The effect of gold nanoring height (*h*) on the extinction spectrum under different refractive indices: (**a**) h = 130 nm, (**b**) h = 170 nm, and (**c**) h = 180 nm.

**Figure 4:**
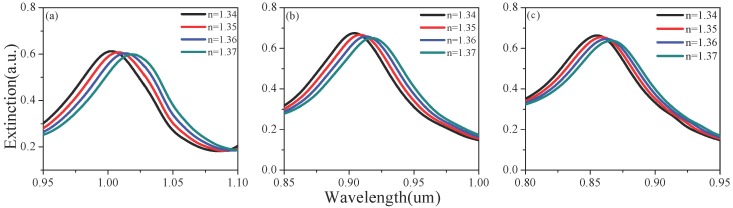
The effect of gold nanoring period (*a*) on the extinction spectrum under different refractive indices: (**a**) a = 300 nm, (**b**) a = 320 nm and (**c**) a = 340 nm.

**Figure 5:**
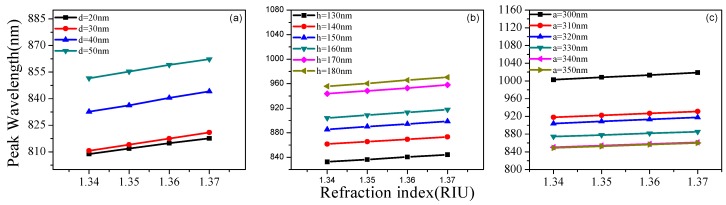
RI sensitivity for different values of (**a**) thickness d, (**b**) height h, and (**c**) period a.

Figure 2 shows the extinction spectra for partial thicknesses d under different background refractive indices. The influence of height h and period a was also analyzed by the same method, which can be seen in Figure 3 and Figure 4. There was a tendency for double formants when h > 170 nm. This is because gold rings may have different couplings at different heights. As the background refractive index increases, the resonance peak will have a red shift. Based on Figure 2, Figure 3 and Figure 4, Figure 5 shows the RI sensitivity for different values of the parameters. Obviously, the RI sensitivity first increased and then decreased with the increase of the thickness, and the maximum RI sensitivity was obtained at d = 40 nm. Moreover, the RI sensitivity decreased as the period a increased. We can therefore draw the conclusion that smaller period and larger height are beneficial to the enhancement of RI sensitivity. That being said, we considered various factors to avoid the double-peak phenomenon when h > 170 nm, obtaining the results listed in Table 2. These results are generally the same as those of the analysis in the previous section, with the RI sensitivity up to 530.4 nm/RIU when selecting d = 40 nm, a = 300 nm, h = 160 nm.

**Figure 6:**
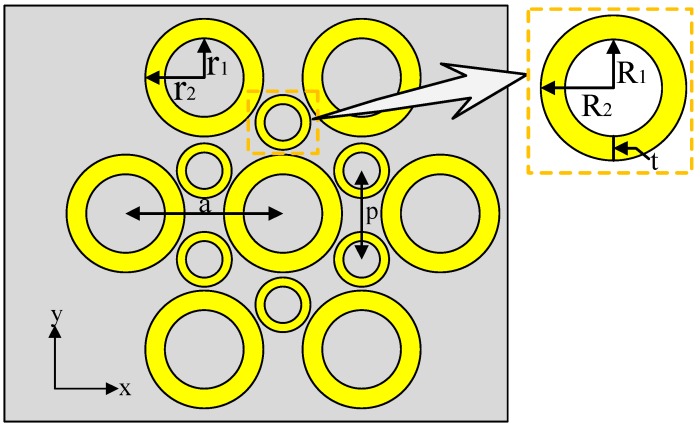
Side view of Structure B.

It was also necessary to further optimize the original design. The side view of the improved structure is shown in Figure 6. A set of smaller gold rings with outer radius of *R*_2_ = 30 nm, inner radius of *R*_1_ = 10 nm, height of *h =* 160 nm, and center distance of $p=\sqrt{3}/3a$ (Structure B) were added into the previous hexagon. The main purpose was to improve the RI sensitivity by increasing the coupling area. The coupling between adjacent LSPRs was enhanced under SPP modulation [25]. The RI sensitivity for Structure B and the increase rates relative to Structure A are shown in Table 3. The increase rate *I* can be obtained by Equation (7), where $RI_{A}$ and $RI_{B}$ are the refractive index sensitivity of Structure A and B, respectively. If Structure A_4_ was replaced by Structure B_4_, its RI sensitivity remained the largest. Meanwhile, the RI sensitivity had the largest growth rate when the structure changed from A_1_ to B_1_. Obviously, the increasing trend of RI sensitivity was basically the same, and this optimization method was effective for sensitivity enhancement.

(7) $$I=\frac{RI_{B}-RI_{A}}{RI_{A}}$$

**Figure 7:**
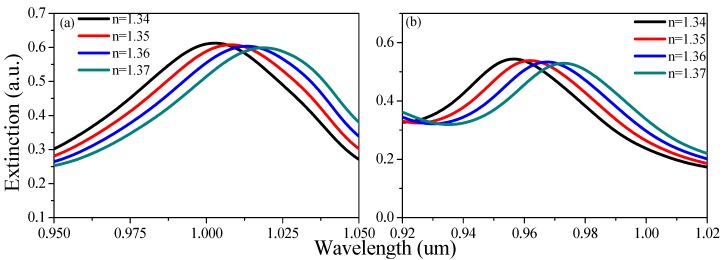
Extinction spectra under different refractive indices for (**a**) Structure A_4_ and (**b**) Structure B_4_.

As an example, Figure 7 indicates the spectral difference between Structure *A_4_* (*d* = 40 nm, *h* = 160 nm, and *a* = 300 nm) and Structure B_4_ (*d* = 50 nm, *h* = 160 nm, *a* = 300 nm, and $p=\sqrt{3}/3a$, t = 20 nm). The RI sensitivity increased from 530.4 nm/RIU to 550.08 nm/RIU. This was because the coupling area between different gold rings increased when new, smaller gold rings were added. When adjacent SPP modulation occurred, the coupling between adjacent LSPRs was enhanced to improve the RI sensitivity. It can also be seen that the absorption rate of the structure decreased to some extent. This was because the increase in density and area of the gold ring made the metal more reflective following the addition of the hexagonal internal gold rings. In particular, such a gold-ring array structure was very convenient for coating a gas-sensitive film onto the inner and outer surfaces of the gold rings. In the next section, we discuss the measurement of methane concentration by the combined use of LSPR effect and gas-sensitive reaction.

## 3. Results and Discussion

**Figure 8:**
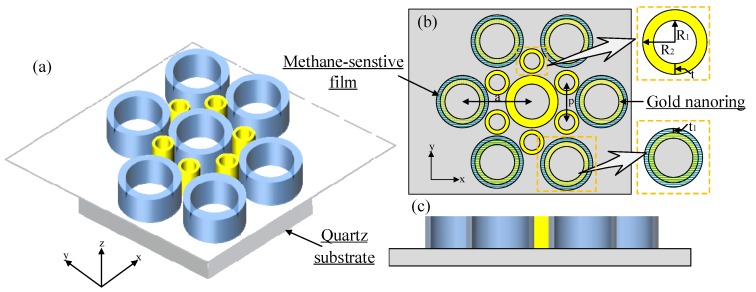
Methane gas sensor structure: (**a**) 3D view, (**b**) top view, and (**c**) side view of Structure *B*.

The gold-ring array structure can be used not only for refractive-index sensing, but also for gas measurement by plating a gas-sensitive film onto the inner surface. Figure 8 describes the methane sensor structure. According to the analysis in Section 2, even though Structure B_4_ had the largest RI sensitivity, the spacing between the bigger gold ring and the smaller gold ring was too small to be coated with methane-sensitive film. Therefore, we chose Structure B_5_ as the sensing platform. At the same time, the sensing performances were compared between Structure A_5_ and Structure B_5_.

**Figure 9:**
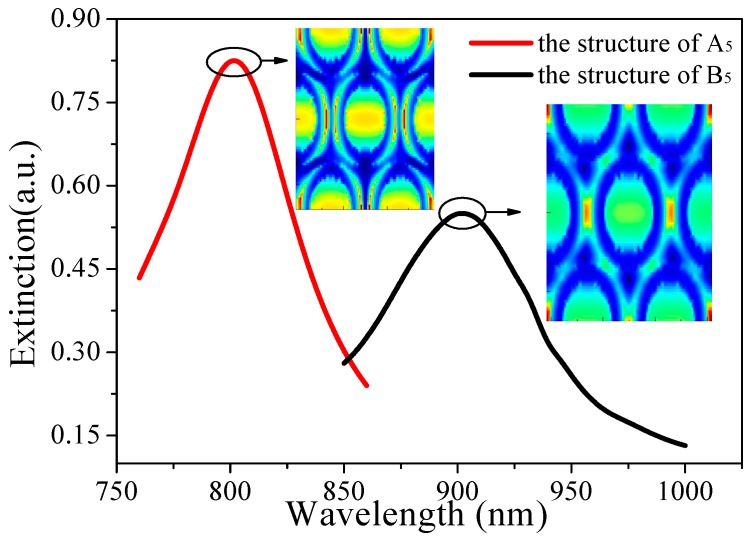
Extinction spectra of Structure A_5_ and Structure B_5_ after coating and electric field distribution of these structures at 802 nm and 902 nm wavelengths.

On the basis of the principle of LSPR in Section 2, the variation of gas concentration led to a change of effective permittivity and a shift of the resonance peak. It can be seen from Figure 9 that Structure A_5_ and Structure B_5_ exhibited strong coupling at 802 nm and 902 nm, respectively. The electric field diagram indicates that the plasmon resonance mode was significantly enhanced at 802 nm and 902 nm. At the same time, the asymmetry of the charge distribution on the inner and outer surfaces of the gold rings showed that the generation of the resonance peak was due not only to the dipole resonance but also to the mode coupling. Since a periodic-swap array has better resonance coupling than a single-loop or non-periodic-loop array, the smaller hexagonal periodic gold ring array can enhance the coupling surface in order to magnify the plasmon resonance effect.

**Figure 10:**
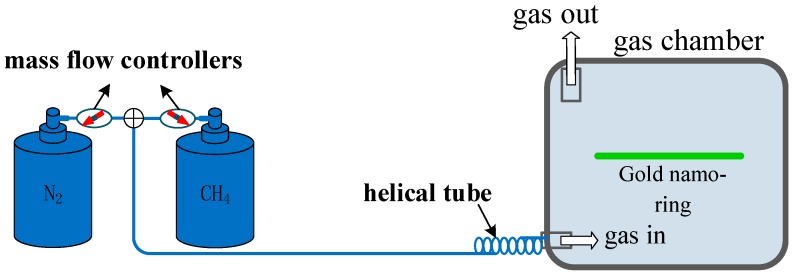
Device for manipulating the gas concentration.

A UV-curable fluoro-silicone (UVCFS) nano-film with the inclusion of cryptophane A was selected as the methane-sensitive film. This film can be fabricated with a capillary dip-coating technique. Moreover, temperature and humidity have minimal effect on methane-sensitive films [26]. The larger gold rings were coated with the methane-sensitive film to a thickness of t_1_ = 8 nm, while the concentration of the target gas was derived from the measured refractive index of the film. The refractive index of methane-sensitive film decreased linearly with an increase in methane concentration within the range of 0–3% [27]. Moreover, for each 1% increase in methane concentration ($C_{CH_{4}}$), the refractive index of methane-sensitive film ($n_{eff}$) decreased by 0.0038 within the range of 1.4478–1.4364, as shown by Equation (8). The gas concentration could be manipulated in the device shown in Figure 10. The methane concentration was precisely controlled by two mass-flow controllers, and the gases were mixed in a stainless steel helical tube between the controllers and the chamber.

(8) $$n_{eff}=1.4478-0.0038C_{CH_{4}}$$

**Figure 11:**
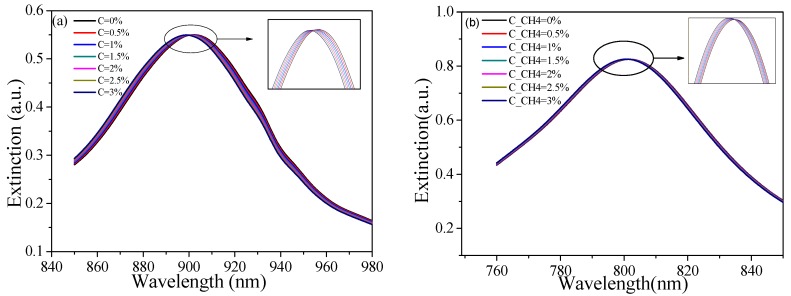
Extinction spectra at different methane concentrations for (**a**) Structure B_5_ and (**b**) Structure A_5_.

**Figure 12:**
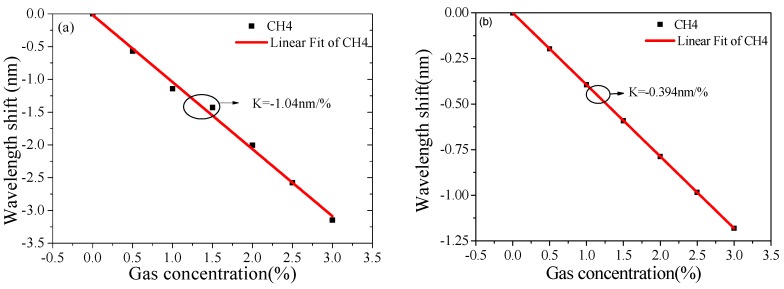
Wavelength shift vs methane concentrations for (**a**) Structure B_5_ and (**b**) Structure A_5_.

Figure 11 shows the extinction spectra at different gas concentrations of Structure A_5_ and Structure B_5_, respectively. With an increase of gas concentration, the resonance wavelength had a linear blue shift; the corresponding sensitivity curves are shown in Figure 12, Table 4 lists the calculated results; the methane sensitivities were about −1.02 nm/% for Structure B_5_ and −0.394 nm/% for Structure A_5_. With verification of the above results, the optimization method used in the previous section was also applicable to the methane measurement here reported. If more target gases need to be measured, it is only necessary to change the related gas-sensitive film. The proposed method is also applicable to quantitative analyses of components concentration and qualitative analyses of gas composition.

## 4. Conclusions

This study proposed an LSPR sensor based on a hexagonal periodic gold ring array. The RI sensitivity can be substantially enhanced by optimizing the design of the sensor structure. After coating a methane-sensitive membrane on the inner surface of the gold rings, this simple sensor structure was also found to be applicable to methane measurement. The maximum RI sensitivity was about 550.08 nm/RIU, while the methane concentration sensitivity reached a maximum of −1.02 nm/%. The combined use of LSPR and a gas-sensitive reaction offers a new method for of gas composition analysis.

## Figures and Tables

**Table 1:** The resonance peak, full width at half maximum (FWHM), and extinction rate for different values of the parameters *d* (thicknesses), *a* (period), *h* (height).

*d* [nm]	*a* [nm]	*h* [nm]	Resonance Peak [nm]	Full Width at Half Maximum (FWHM) [nm]	Extinction Ratio %
20	400	160	760	87	83.1
30	400	160	731	80	80.5
40	400	160	737	72	80.2
50	400	160	743	79	81.9
40	310	160	778	88	87.2
40	320	160	772	73	89.2
40	330	160	763	76	93.7
40	340	160	731	80	90
40	320	140	734	78	92.9
40	320	150	753	64	97.2
40	320	160	772	73	87.2
40	320	170	802	76	74

**Table 2:** RI sensitivity of different parameters at *d* = 40 nm.

Group Number	Period	Height	Sensitivity
	a	h	[nm/RIU]
$A_{1}$	300	150	449.87
$A_{2}$	320	150	441.05
$A_{3}$	340	150	370.2
$A_{4}$	300	160	530.4
$A_{5}$	320	160	453.66
$A_{6}$	340	160	372.9
$A_{7}$	300	170	449.72
$A_{8}$	320	170	473.63
$A_{9}$	340	170	412.4

**Table 3:** RI sensitivity for Structure B and comparison with Structure A.

Group Number	Period	High	Thickness	Sensitivity	Increase Rate
	a	h	*t* [nm]	[nm/RIU]	[%]
$B_{1}$	300	150	20	533.24	18.5
$B_{2}$	320	150	20	509.22	15.5
$B_{3}$	340	150	20	390.9	5.6
$B_{4}$	300	160	20	550.08	3.7
$B_{5}$	320	160	20	532.71	17.4
$B_{6}$	340	160	20	398.53	6.9
$B_{7}$	300	170	20	486.06	8.1
$B_{8}$	320	170	20	528.6	11.6
$B_{9}$	340	170	20	457.86	11

**Table 4:** Values of the calculated results.

Type of Gas	The Structure	The Sensitivity of Gas [nm/%]	Increase Rate [%]
CH_4_	*B*_5_	−1.04	159
	*A*_5_	−0.394	
